# Serum proteins and paraproteins in women with silicone implants and connective tissue disease: a case–control study

**DOI:** 10.1186/ar2295

**Published:** 2007-09-17

**Authors:** Gyorgy Csako, Rene Costello, Ejaz A Shamim, Terrance P O'Hanlon, Anthony Tran, Daniel J Clauw, H James Williams, Frederick W Miller

**Affiliations:** 1 Department of Laboratory Medicine, Clinical Center, NIH, DHHS, 9000 Rockville Pike, Bethesda, MD 20892, USA; 2 Environmental Autoimmunity Group, National Institute of Environmental Health Sciences, NIH, DHHS, 9000 Rockville Pike, Bethesda, MD 20892, USA; 3 Association of Public Health Laboratories, 8515 Georgia Avenue, Suite 700, Silver Spring, MD 20910, USA; 4 Division of Rheumatology, Department of Medicine, University of Michigan Medical School, 101 Simpson Drive, Ann Arbor, MI 48109, USA; 5 Division of Rheumatology, Department of Internal Medicine, University of Utah Medical Center, 50 North Medical Drive, Salt Lake City, UT 84132, USA

## Abstract

Prior studies have suggested abnormalities of serum proteins, including paraproteins, in women with silicone implants but did not control for the presence of connective-tissue disease (CTD). This retrospective case–control study, performed in tertiary-care academic centers, assessed possible alterations of serum proteins, including paraproteins, in such a population. Seventy-four women with silicone implants who subsequently developed CTD, and 74 age-matched and CTD-matched women without silicone implants, were assessed in the primary study; other groups were used for additional comparisons. Routine serum protein determinations and high-sensitivity protein electrophoresis and immunofixation electrophoresis were performed for detection of paraproteins. Women with silicone implants, either with or without CTD, had significantly lower serum total protein and α_1_-globulin, α_2_-globulin, β-globulin, γ-globulin, and IgG levels compared with those without silicone implants. There was no significant difference, however, in the frequency of paraproteinemia between women with silicone implants and CTD (9.5%) and age-matched and CTD-matched women without silicone implants (5.4%) (odds ratio, 1.82; 95% confidence interval, 0.51–6.45). Paraprotein isotypes were similar in the two groups, and the clinical characteristics of the 13 women with paraproteinemia were comparable with an independent population of 10 women with silicone breast implants, CTD, and previously diagnosed monoclonal gammopathies. In summary, this first comprehensive study of serum proteins in women with silicone implants and CTD found no substantially increased risk of monoclonal gammopathy. Women with silicone implants, however, had unexpectedly low serum globulin and immunoglobulin levels, with or without the subsequent development of CTD. The causes and clinical implications of these findings require further investigation.

## Introduction

Local adverse effects from silicone implants, which include surgically placed devices as well as injections of liquid silicone, are well recognized [1,2], but systemic effects are not supported by current studies. Systematic reviews [3,4] and four meta-analyses including data from up to 20 retrospective cohort, case–control, and cross-sectional studies [5-8] have failed to find significantly increased risks of any CTD after receiving silicone implants.

Few studies, however, have evaluated serum proteins and paraproteins in women with silicone implants. Plasmacytomas have been induced with silicone gel from breast implants in susceptible mouse strains [9], and several uncontrolled clinical reports suggested that silicone implants might be associated with multiple myeloma (MM) and its possible precursor, monoclonal gammopathy of undetermined significance (MGUS) [10-12]. One investigation evaluated the risk for MGUS in a retrospective case–control study of women with and without silicone implants, and found a nonsignificant increase (odds ratio, 1.25; 95% confidence interval, 0.27–6.39) [13]. Another case–control study found no increase in immunoglobulin levels or other immunologic parameters, with the exception of anti-single-stranded DNA autoantibodies, in women with silicone implants [14]. None of these studies, however, assessed the role of concomitant CTD, which has been reported to be a risk factor for monoclonal immunoglobulins (paraproteins) and is associated with MGUS in 3–6% of cases [15].

The possible increased risk of paraproteins in women with silicone implants and CTD, as well as the limited information on other serum proteins in this population, prompted us to assess whether silicone implants in women who subsequently developed CTD are associated with altered serum protein profiles and/or a higher prevalence of serum paraproteins.

## Materials and methods

### Patients and study design

All patients were enrolled prospectively in studies of the pathogenesis of the diseases described, and extensive clinical information was collected at enrollment to ensure subjects met the diagnostic criteria. The current study was retrospective in that subjects enrolled in the prior studies were identified based on the presence of a stored serum sample.

The primary study population (Group 1) included 74 women who developed CTD after receiving silicone implants. Group 1 were enrolled in studies of the pathogenesis of CTD developing after receiving silicone implants at the US Food and Drug Administration (FDA) and the National Institutes of Health (NIH) from 1993 to 2000. These subjects were matched to 74 age-matched and CTD-matched women without silicone implants (Group 2) enrolled in other protocols at the FDA and NIH between 1993 and 2000, and subjects from a study of the underlying mechanisms of primary fibromyalgia (fibromyalgia syndrome (FMS)) from 1986 to 1989 and from the Early Undifferentiated Connective Tissue Disease study as part of the Cooperative Systematic Studies of the Rheumatic Diseases enrolled between 1982 and 1987.

We also matched 14 women with silicone implants but no CTD (Group 3) to 14 women without silicone implants or CTD (Group 4) for exploratory evaluations of the effects of silicone implants without CTD. In other exploratory analyses, cases from Group 2 that were found to have paraproteins were compared with those paraprotein cases in independent groups of 28 women with CTD but without silicone implants (Group 5), and were compared with 10 women with CTD and previously diagnosed gammopathies following silicone breast implants (Group 6). Apart from Group 6, none of the women had been diagnosed with paraproteinemia previously.

All women gave informed consent to allow their clinical information and serum to be used for research purposes in clinical studies approved by institutional review boards at the FDA, at the NIH, at Georgetown University, Washington, DC, and at the University of Utah, Salt Lake City, UT.

### Disease classification criteria

Clinical diagnoses for CTD were determined following American College of Rheumatology criteria or were based upon proposed criteria when American College of Rheumatology criteria were not available for a given condition. Patients with the following diseases were included in the analyses: definite or probable polymyositis or dermatomyositis [16], systemic sclerosis (scleroderma) [17], systemic lupus erythematosus [18], FMS [19], and undifferentiated connective tissue disease (UCTD) and unexplained polyarthritis [20]. Disease duration was defined as the time between onset of disease and specimen collection.

### Selection of women with silicone implants for the case–control study

As already described, 88 women (Groups 1 and 3) enrolled into FDA and NIH protocols investigating the pathogenesis of CTD following silicone implants were chosen for study on the basis of the presence of documented silicone implants and an available serum sample collected for research purposes. Silicone implants consisted of surgically placed devices and liquid silicone injections. The most common types of silicone implants were breast implants (*n *= 81); these included silicone gel-filled devices (*n *= 68), saline implants (*n *= 6), both polyurethane and silicone gel implants (*n *= 3), and both polyurethane and saline implants (*n *= 4). Two women with breast implants also had additional silicone implants (one had liquid silicone injections and one had bilateral cheek implants). Five women received only silicone cheek implants. The time of silicone exposure was defined as the time from placement or injection of the first implant to specimen collection. Silicone implant duration was defined as the time from placement of the first implant to removal of the last implant.

### Selection of women without silicone implants for the case–control study

Women without silicone implants consisted of subjects enrolled into investigations into the natural history of CTD diseases conducted at the FDA and the NIH, of the pathogenesis of FMS at Georgetown University and of women enrolled in a multicenter inception cohort of early UCTD who were followed to assess ultimate clinical outcomes [20].

The 88 women with silicone implants (Groups 1 and 3) were randomly matched for age and specific CTD (or, as appropriate, for lack of CTD) with women from these populations without silicone implants (Groups 2 and 4). Seventy-five patients (85%) were matched within 5 years of age, 12 patients (14%) were matched within 6–10 years, and one patient (1%) was matched within 15 years of age. The mean age was 50.2 ± 8.8 years (median 50 years, range 30–76 years) in the silicone implants group versus 49.4 ± 8.4 years (median 50 years, range 32–71 years) in those without silicone implants (*P *= 0.06).

The women were also matched for diagnosis (64 patients with various inflammatory CTD, 10 patients with noninflammatory CTD (FMS), and 14 patients without any CTD in both groups). Except for the single systemic lupus erythematosus subject with silicone implants, who was matched with a dermatomyositis subject without silicone implants, all matched subjects shared the same clinical diagnoses. Diagnostic categories (and the total number of matched women) included UCTD (*n *= 78), polymyositis/dermatomyositis (*n *= 27), FMS (*n *= 20), systemic sclerosis (*n *= 20), unexplained polyarthritis (*n *= 2), systemic lupus erythematosus (*n *= 1), and no CTD (*n *= 28). The frequencies of UCTD criteria in the silicone group were: unexplained polyarthritis, 72%; myalgias, 59%; isolated keratoconjunctivitis sicca, 38%; Raynaud's disease, 31%; rash, 31%; central nervous system symptoms, 13%; pulmonary symptoms, 5%; elevated erythrocyte sedimentation rate, 5%; false-positive serologic test for syphilis, 5%; and peripheral neuropathy, 3%.

Although attempts were made to race match whenever possible, there were more African-Americans and Hispanics in women without silicone implants (69 Whites, 14 Blacks, three Hispanics, one Oriental, one unknown race) than in those with silicone implants (87 Whites, one Hispanic).

### Determination of serum total protein and immunoglobulins

Serum samples were stored at -80°C until analysis and laboratory personnel were blinded to the group identity of the samples. Total serum protein was measured by a biuret method on Hitachi 917 automated chemistry analyzers (Roche Diagnostics, Indianapolis, IN, USA). Serum IgG, IgA, and IgM levels were quantified by immunonephelometry on a protein array automated immunochemistry analyzer (Beckman-Coulter, Brea, CA, USA).

### Serum protein electrophoresis

For quantification of various protein fractions and paraprotein bands, all sera first underwent electrophoresis in agarose gel by a semi-automated electrophoretic system (SPIFE™ SPE Vis-60; Helena Labs, Beaumont, TX, USA). After staining with amido black, gels were scanned with an EDC densitometer (Helena Labs) at 545 nm. As part of the immunofixation electrophoretic screen for paraproteins (see below), all sera also underwent electrophoresis in agarose gel (Hydragel; Sebia, Norcross, GA, USA) by another semi-automated electrophoretic system (Hydrasys; Sebia, Norcross, GA, USA). This screen involved the use of a more sensitive protein stain (acid violet) for improved detection of paraproteins.

### Serum immunofixation electrophoresis

After electrophoresis of the sera in agarose gel (Hydrasys; Sebia, Norcross, GA, USA), immunofixation was performed first with a mixture of antibodies (anti-α, anti-γ, and anti-μ heavy chains, and anti-κ and anti-λ free light chains) (Penta screen; Sebia, Norcross, GA, USA). Patterns were visualized by staining with a highly sensitive protein stain (acid violet). All patterns considered positive or suggestive for the presence of paraproteins prompted full immunofixation electrophoresis work-up using the Hydrasys with acid violet staining. Two-thirds of the specimens with positive findings for paraprotein(s) were also confirmed by a conventional manual immunofixation electrophoresis method involving the use of Paragon Blue stain (Paragon; Beckman-Coulter, Brea, CA, USA).

### Statistical analyses

Data are shown as the mean ± standard deviation. Statistically significant differences between groups were assessed with a paired *t *test or the McNemar test as deemed appropriate. The odds ratio and 95% confidence interval were calculated by standard methods. All *P *values are two-tailed, and *P *< 0.05 was considered significant.

## Results

### Characteristics of women in the case–control study with connective tissue disease

The time between placement of the first silicone implants and collection of the blood specimen for testing ('silicone exposure') was 15.4 ± 7.3 years (median 15.8 years) for the 86 assessable cases, and the implant duration was 12.8 ± 5.6 years (median 12.9 years) for the 48 assessable cases. Of women with silicone implants who were available for assessment (*n *= 63), 52% (33/63) probably had implant rupture based on magnetic resonance imaging (*n *= 5) or signs and symptoms suggestive of rupture, such as the acute development of pain in the implant site or sudden changes in the size, shape, or consistency of the implant (*n *= 27), of which 17 ruptures were documented at surgery. Silicone breast implants were removed at least once in 67% of the 74 cases who were available for assessment: once in 55% of these cases, twice in 5% of these cases, and three times in 7% of these cases. The mean ± standard deviation duration of CTD was 6.8 ± 6.6 years (median 4.2 years) for the 64 assessable cases in Group 1.

### Serum protein profiles

**Figure 1:**
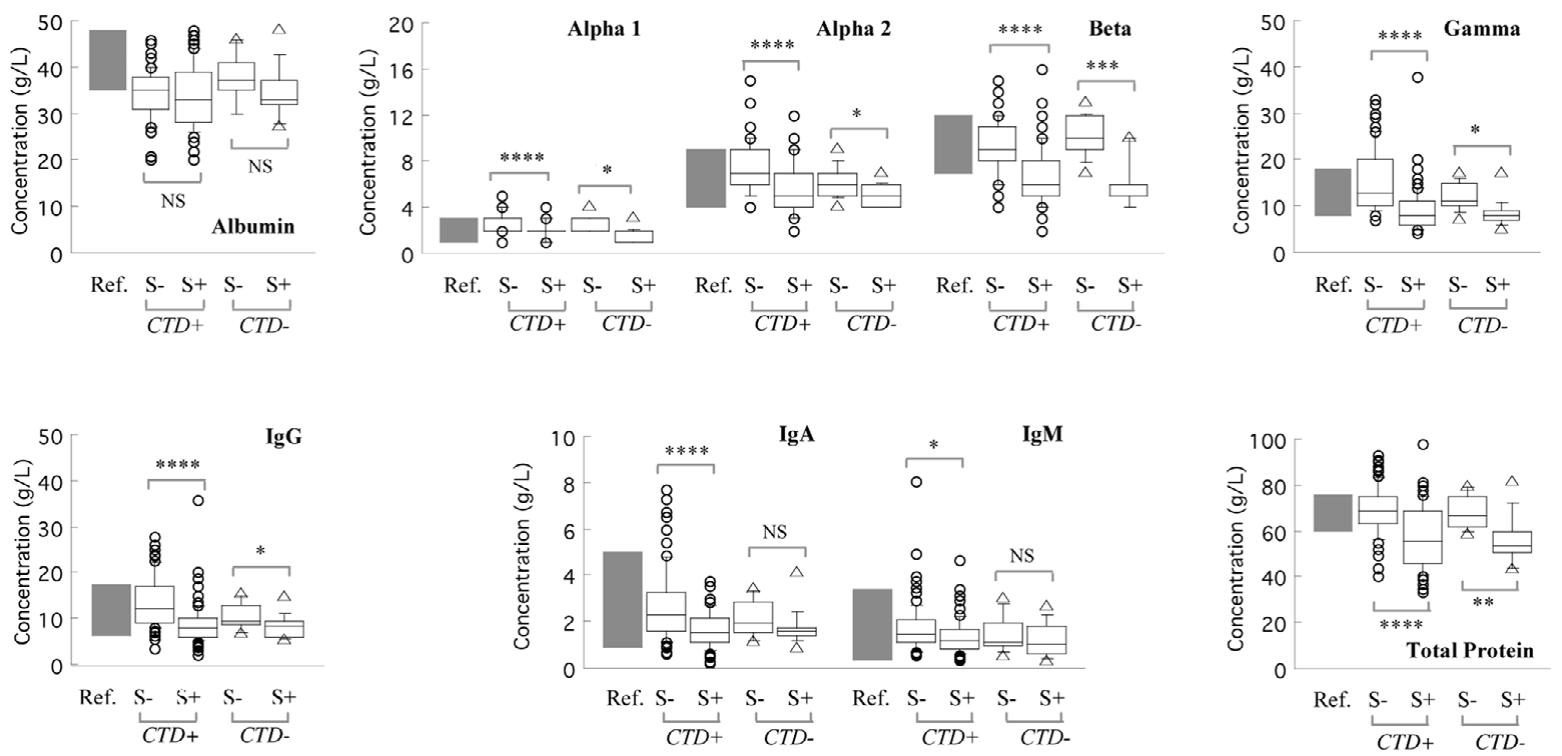
Serum proteins and immunoglobulins in women with/without connective tissue disease and with/without silicone implants. Box plots: vertical lines identify the 10th and 90th percentiles, horizontal lines in boxes identify the 25th, 50th (median), and 75th percentiles, while 'outliers' are shown with open symbols. Ref. (filled columns), reference intervals; CTD+ and S-, Group 2 (74 women with connective tissue disease (CTD) but no silicone implants); CTD+ and S+, Group 1 (74 women with CTD and with silicone implants); CTD- and S-, Group 3 (14 women with no disease and no silicone implants); CTD- and S+, Group 4 (14 women with no disease but silicone implants). Alpha 1, α_1_-globulin; alpha 2, α_2_-globulin; beta, β-globulin; gamma, γ-globulin. NS, not significant; *P < 0.05, **P < 0.01, ***P < 0.001, and ****P < 0.0001 by paired *t *test.

Regardless of the presence or absence of CTD, there was a trend toward lower levels of total protein and various globulin fractions in women with silicone implants (Figure 1). The total protein, all globulin fractions (α_1_-globulin, α_2_-globulin, β-globulin, and γ-globulin), and IgG levels were significantly lower (*P *< 0.05) in women with silicone implants compared with those without silicone implants in both the presence and absence of CTD (Figure 1). IgA and IgM levels were also significantly lower (*P *< 0.05) in women with silicone implants who developed CTD (Group 1) compared with matched women without silicone implants who developed CTD (Group 2). These differences were less often significant in women without CTD (Group 3 versus Group 4), although these samples sizes were smaller. Only albumin failed to show significantly lower levels with silicone implant exposure either in the presence or absence of CTD. Apart from fewer significant differences between groups of women with FMS, the results were also similar when women with/without silicone implants were compared in subsets of inflammatory CTD (64 pairs) and FMS (10 pairs) from Groups 1 and 2 (data not shown).

### Serum paraproteins

No paraproteins were found in women without CTD either in the presence (Group 3) or absence of silicone implants (Group 4). Paraproteins were also relatively uncommon in the sera of women with CTD, either with silicone implants (Group 1) or without silicone implants (Group 2) (Table 1). Full immunofixation electrophoresis workups revealed no significant difference in paraproteinemia rates between women with silicone implants (7/74 or 9.5%) (Table 2) and without silicone implants (4/74 or 5.4%) (Table 3) (odds ratio, 1.82; 95% confidence interval, 0.51–6.45). Five of the seven women with CTD, silicone implants and paraproteinemia had UCTD but there were no significant differences (*P *= 0.55) in the CTD diagnostic distribution patterns between these women with and without silicone implants (Table 1).

**Table 1:** Serum paraproteins in the case–control study of 74 age-matched and connective tissue disease (CTD) diagnosis-matched women with and without silicone implants

	Silicone implant	
	
	Yes (Group 1)	No (Group 2)
Age (years)^a^	49.9 ± 8.7	49.8 ± 8.4
Number of women with paraprotein band(s)/total^b^	7/74 (9.5%)	4/74 (5.4%)
Number of women with paraprotein band(s) according to primary CTD diagnosis		
Unexplained polyarthritis	0/1 (0%)	0/1 (0%)
Systemic sclerosis	1/10 (10%)	0/10 (0%)
Systemic lupus erythematosus	0/1 (0%)	0/0 (0%)
Polymyositis/dermatomyositis	1/13 (4%)	2/14 (4%)
Undifferentiated CTD	4/39 (13%)	2/39 (5%)
Fibromyalgia syndrome	1/10 (0%)	0/10 (10%)
Type of paraprotein band(s)^c^		
IgG(κ)	4	5
IgG(λ)	1	1
IgM(κ)	0	0
IgM(λ)	2	1

^a^Presented as the mean ± standard deviation, no significant difference by paired *t *test (*P *= 0.82). ^b^No significant differences between Groups 1 and 2 using the McNemar test (*P *= 0.55). ^c^One woman with dermatomyositis had two IgG(κ) and one IgG(λ) bands, whereas a patient with polymyositis had both IgG(κ) and IgM(λ) bands.

**Table 2:** Characteristics of women with connective tissue disease and silicone implants in whom paraproteinemia was identified from Group 1

Age (years)	Connective tissue disease		Silicone implant				Serum paraprotein (isotype)^a^
		
	Type	Duration (years)	Type	Duration (years)	Exposure (years)	Rupture^b^	
48	UCTD	15.4	Silicone gel, saline	17.7	18.0	Yes	IgM(λ)
49	FMS/UCTD	15.2	Silicone gel (chin)	6.0	17.7	Yes	IgG(κ)
53	UCTD	6.2	Saline	3.5	3.5	Yes	IgG(λ)
55	Systemic sclerosis	12.1	Polyurethane	4.3	16.1	n/a	IgG(κ)
60	UCTD/FMS	3.9	Silicone gel	20.1	24.2	Yes	IgG(κ)
61	Dermatomyositis	4.2	Silicone gel	10.0	10.2	Yes	IgG(κ)
63	UCTD	16.1	Silicone gel	n/a	23.3	Yes	IgM(λ)
All: (55.6 ± 5.9)		10.4 ± 5.5		10.3 ± 7.1	16.1 ± 7.3		

Subjects are listed in increasing order of age. All subjects were White. FMS, fibromyalgia syndrome; UCTD, undifferentiated connective tissue disease; n/a, not available. ^a^Based on protein electrophoresis, all paraprotein bands were considered weak (defined as <1 g/l). ^b^Implant rupture was suspected by signs and symptoms in six cases and was documented at surgery in four cases.

**Table 3:** Characteristics of women with connective tissue disease but without silicone implants in whom paraproteinemia was identified from Group 2 and Group 5

Age (years)	Ethnicity	Connective tissue disease	Serum paraprotein (isotype)^a^
*32*	*Hispanic*	*Undifferentiated connective tissue disease*	*IgG(κ)*
*43*	*White*	*Polymyositis*	*IgG(κ)*
*47*	*Hispanic*	*Undifferentiated connective tissue disease*	*IgG(κ)*
55	White	Dermatomyositis	IgG(κ) x2, IgG(λ)
56	Black	Polymyositis	IgG(κ), IgM(λ)
65	White	Undifferentiated connective tissue disease	IgG(κ)
All: 49.7 ± 11.6			
Group 2 patients only (*n *= 4): 55.8 ± 7.4			

Subjects are listed in increasing order of age and the first 3 cases (italicized) are from Group 5. ^a^Based on protein electrophoresis, all paraprotein bands were considered weak (defined as <1 g/l).

There was only a single case of paraproteinemia in women with FMS, and this case occurred in a subject with a silicone implant (Table 1). Because of the concern that only inflammatory CTD might be associated with paraproteins, we also compared only the non-FMS cases. After omitting the 10 pairs of cases of FMS from the analysis, there was no significant difference in the frequency of paraproteins between the remaining 64 women with inflammatory CTD and silicone implants (six cases) and the matched 64 women with inflammatory CTD without implants (four cases). Of interest, many women with silicone implants and paraproteinemia probably had implant rupture (Table 2). While the women with silicone implants and paraproteinemia (Table 2) were older than those women with paraproteinemia without silicone implants (Table 3), the difference did not reach statistical significance (*P *= 0.26) and the age difference completely disappeared when only women from Groups 1 and 2 were compared. Within the group of women with silicone implants and CTD (Group 1), between those women with paraproteins and those women without paraproteins there were only borderline significant differences or no significant differences (Mann–Whitney U test) in age (55.6 years versus 49.3 years, *P *= 0.041), in duration of CTD (10.4 versus 6.4 years, *P *= 0.051), in implant duration (10.2 years versus 12.5 years, *P *= 0.267), and in duration of silicone exposure (16.1 years versus 15.4 years, *P *= 0.783). After omitting the 10 cases of FMS, the remaining 64 pairs of women with inflammatory CTD exhibited similar patterns (data not shown). Furthermore, irrespective of including or excluding cases of FMS, there were no significant differences in age between those women with and those women without paraproteins within the group of women with CTD but no silicone implant (Groups 2 and 5) (data not shown).

The paraprotein isotypes were similar in women with and without silicone implants, and included IgG(κ), IgG(λ), and IgM(λ) (Table 1). While every subject with paraproteinemia in the silicone implant group had only a single band, however, this was not true in the comparison group without silicone implants. In this latter group, one woman with dermatomyositis had two IgG(κ) bands and one IgG(λ) band, and a woman with polymyositis had both IgG(κ) and IgM(λ) bands. The total number of paraprotein bands was consequently the same in the two study groups (Table 1). All paraproteins occurred in low concentrations. Only an IgG(κ) band was quantifiable (estimated serum concentration from the protein electrophoretic pattern, ≃1 g/l); all others had trace quantities (<1 g/l estimated concentration from protein electrophoresis) (Table 2). No subject with paraprotein(s) developed multiple myeloma or other malignancy during the 2-year follow-up period after enrollment in the study.

### Comparison of women with paraproteinemia in other study groups

To assess whether the paraproteinemia cases identified in the case–control studies were different from those in independent populations, women with paraproteins in the case–control studies were compared with those from another group of 28 women with CTD without silicone implants (Group 5), and were compared with 10 women with silicone implants and previously diagnosed paraproteins (Group 6) (Table 4).

**Table 4:** Characteristics of women with connective tissue disease and silicone breast implants who were previously diagnosed with paraproteinemia (Group 6)

Age (years)	Connective tissue disease		Silicone implant				Serum paraprotein (concentration and isotype)^a^
		
	Type	Duration (years)	Type	Duration (years)	Exposure (years)	Rupture^b^	
43	UCTD/FMS	7.0	Silicone gel	14.2	17.1	Yes	6 g/l IgG(κ)
44	UCTD	12.8	Silicone gel	15.3	17.7	No	3 g/l IgG(λ), 3 g/l IgG(λ)
46	UCTD	7.1	Silicone gel	15.1	17.2	Yes	4 g/l IgG(κ), IgG(λ)
50	UCTD	4.0	Silicone gel/polyurethane	10.5	17.4	Yes	5 g/l IgM(κ)
50	UCTD	8.0	Silicone gel	n/a	19.2	Yes	13 g/l IgG(κ), 6 g/l IgG(κ)
52	UCTD	4.6	Silicone gel	14.1	20.5	Yes	4 g/l IgG(κ)
54	UCTD	3.0	Silicone gel/saline	11.4	14.2	Yes	1 g/l IgG(κ)
55	UCTD/FMS	5.0	Silicone gel	21.0	30.0	Yes	7 g/l IgG(λ)
56	UCTD	10.4	Silicone gel	15.1	23.3	No	9 g/l IgG(κ)
58	UCTD	9.3	Silicone gel	13.5	18.1	Yes	22 g/l IgG(λ)
All: 50.8 ± 5.2		7.1 ± 3.1		14.5 ± 3.0	19.5 ± 4.4		

Subjects are listed in increasing order of age. All subjects were White. FMS, fibromyalgia syndrome; UCTD, undifferentiated connective tissue disease; n/a, not available. ^a^Weak paraprotein bands (defined as <1 g/l) are shown without specifying their concentration; all others are shown by concentrations estimated from protein electrophoresis. ^b^Implant rupture was suspected by signs and symptoms in eight cases and was documented at surgery in five cases.

Since the two cases of paraproteinemia identified from Group 5 were comparable in age, paraprotein type, and frequency (2/28 or 7.1%) with those identified from the CTD patients with no silicone implants in the case–control study (Group 2), they were combined for further analysis (Table 3). This group of six women with CTD and paraproteinemia but no silicone implants was similar in age (mean 49.7 years) to the 10 women with CTD, silicone implant exposure, and previously diagnosed paraproteinemia (MGUS or MM) (Group 6, mean age 50.8 years) (Table 4). The seven women with CTD, silicone implant exposure, and paraproteinemia, however, were older (mean 55.6 years) than either of the previous groups (Table 2). The paraprotein types identified in these women (Table 2) were similar to the other two groups of women with paraproteinemia (Tables 3 and 4), and UCTD was the most common clinical diagnosis associated with paraproteinemia in all three groups.

**Figure 2:**
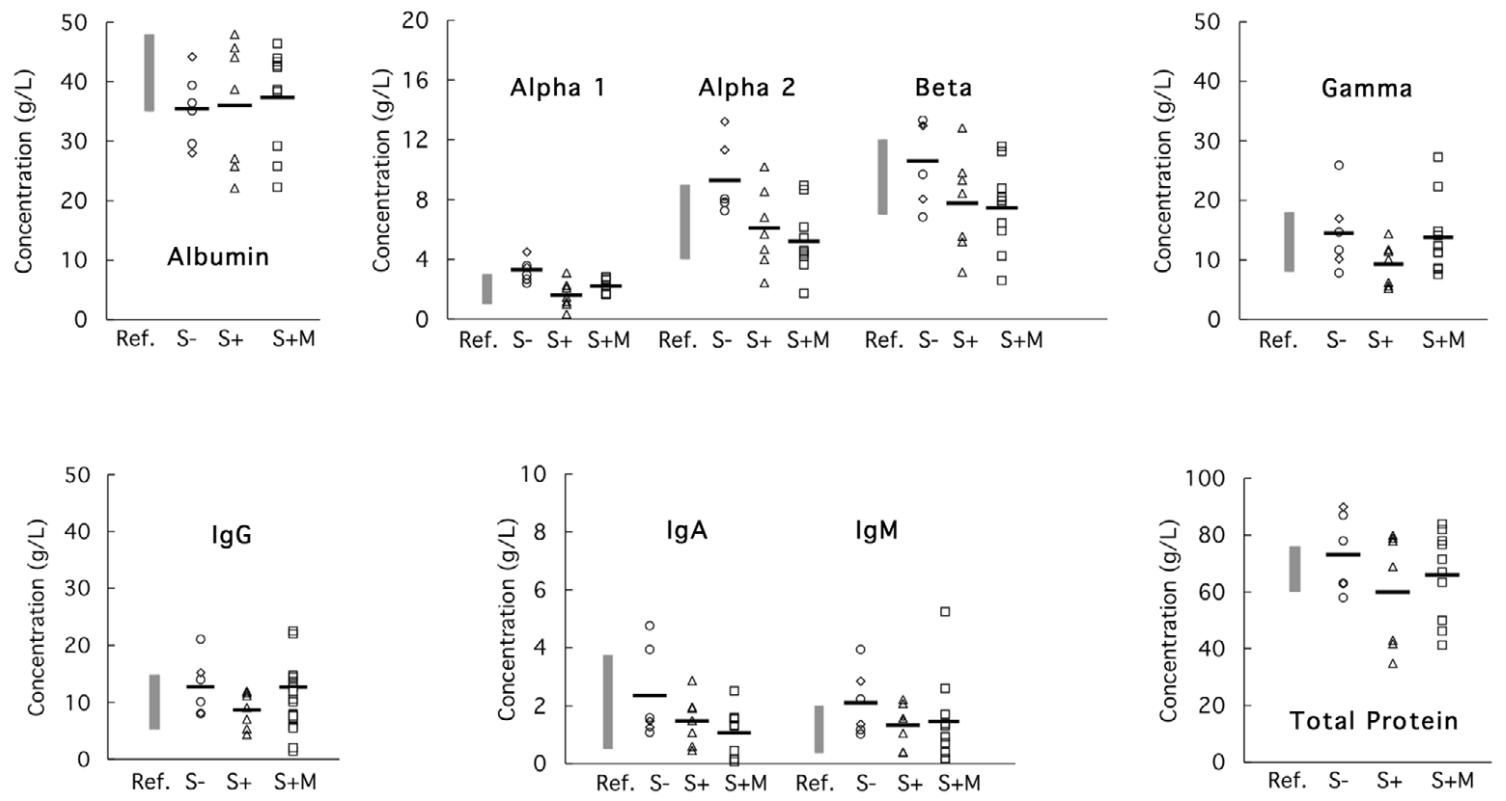
Serum proteins and immunoglobulins in women with connective tissue disease and paraproteinemia with/without silicone implants. Ref. (filled columns), reference intervals; S- (open circles), six women from Groups 2 and 5 with connective tissue disease (CTD) but no silicone implants (see Table 3); S+ (open triangles), seven women from Group 1 with CTD and silicone implants (see Table 2); and S+M, (open rectangles), 10 women (Group 6) with CTD, silicone breast implants, and previously identified paraproteinemia (see Table 4). Alpha 1, α_1_-globulin; alpha 2, α_2_-globulin; beta, β-globulin; gamma, γ-globulin.

Serum protein profiles for women with paraproteinemia revealed similar albumin levels in all three groups. Except for IgG and γ-globulin levels in the previously diagnosed MGUS/MM group with CTD and silicone breast implants (Group 6), various protein fractions tended to be lower in those with silicone implants than in those without silicone implants (Figure 2).

## Discussion

The limited number of studies of serum proteins and paraproteins in women with silicone implants, and the lack of a controlled study taking into account CTD as an additional possible risk factor for serum protein abnormalities, prompted this case–controlled investigation. Paraproteinemia is most often associated with MGUS, which in turn may be a precursor of MM, macroglobulinemia, amyloidosis, or related diseases [21]. Subjects with MGUS often have autoantibodies [22] or autoimmune manifestations [23], and subjects with rheumatic diseases are reported to have higher rates of MGUS [15]. A critical aspect of our case–control study design was therefore to match subjects not only by age, but also by CTD diagnosis to minimize possible confounding. We also used highly sensitive agarose gel electrophoretic and immunofixation electrophoresis methods to maximize detection of serum paraproteins. Because it is possible that FMS patients may differ from inflammatory CTD in the risk of paraproteinemia, we analyzed the women in each group without FMS and found that excluding them does not alter the primary findings of the study.

Although serum paraproteins occurred somewhat more frequently in our study of 74 women with CTD and silicone implants compared with those having CTD without silicone implants (9.5% versus 5.4%), the difference was not statistically significant. Furthermore, since all paraproteins occurred at very low serum concentrations (≤1 g/l), our cases probably represent MGUS [24]. Without additional testing (including bone marrow biopsy, urinary free light-chain assessment, chromosomal studies, and bone surveys) and without follow-up regarding the persistence of paraprotein bands [21,23-28], we could not completely rule out an ongoing malignant process. Nevertheless, none of the subjects reported progression to MM or other hematologic malignancies for up to 2 years after study enrollment. Overall, our findings do not support a major role for silicone implants in inducing monoclonal gammopathies in humans and are consistent with conclusions of prior investigations of silicone implants and MGUS [13,14] or MM [29-31].

We observed higher prevalence of paraproteinemia in women with CTD both with and without silicone implants (8.0% and 4.5%, respectively) than those reported for similarly aged women with any type of breast implants (1.4–1.7%) in one study [14]. Our prevalence rates of paraproteinemia, however, were lower than those reported by the same authors for similarly aged women with breast implants (10.4–15.8%) in another study [16]. MGUS is known to increase in prevalence with age, but our observed prevalence rates are higher than those reported in the literature for 'healthy' adult subjects/populations with ages up to the 70 s (0.5–3.0%) [32-34]. Our finding of three to five times higher prevalence of serum paraproteins over those expected for our age group in the case–control study is, in part, probably related to the combined use of both a highly sensitive protein stain and highly sensitive immunofixation electrophoresis. Using similar analytical methods (Helena agarose electrophoresis and Sebia immunofixation electrophoresis), Kyle and colleagues [35] recently reported a relatively high (3.2%) prevalence of MGUS in a population-based study of 21,463 subjects ≥50 years of age. The age-adjusted rates of MGUS were significantly higher in men than in women (4.0% versus 2.7%) and the prevalence of MGUS increased with age to 5.3% in subjects ≥70 years old. Since these rates are approximately twice that observed earlier, they suggest that the screening methods used in many previous studies were less sensitive than current techniques [35]. In addition to using highly sensitive detection techniques, the high prevalence of paraproteinemia in our study may also be related to the reportedly high prevalence (~3–6%) of paraproteins in subjects with CTD [15] and the relatively high prevalence of CTD (6%) in subjects with MGUS [22]. Regardless of silicone implant exposure, all of our newly identified paraproteinemia cases occurred in women with CTD, resulting in an overall 7.4% prevalence in this group (13/176).

While the total number of cases with paraproteinemia was small in our study, we also observed biclonal cases more often (15% or two out of 13 paraproteinemia cases) than expected from previous investigations in the general population (~2% of MGUS cases) [31,36,37]. Interestingly, both of our biclonal cases occurred in myositis subjects with no silicone implants. The distribution of heavy chain types of the serum paraproteins identified in our study for women with silicone implants and CTD in all 13 cases combined was similar to that we found in women with silicone implants, CTD, and previously diagnosed paraproteins, and similar to that in those described in earlier MGUS case series (71–73% versus 83% for IgG, and 11–14% versus 17% IgM in our population) [13,14,31,36]. We found no IgA paraprotein (0% versus 11–14% in earlier reports for MGUS [31,36]) but this may be due to our comparatively small sample size.

The etiology of MGUS and MM is poorly understood, but case reports and epidemiological studies have shown an increasedassociation with chronic inflammatory conditions [12,38]. Autonomous growth with clonal B-cell expansion and selection mediated by chronic antigen stimulation have been hypothesized to contribute to the development of MM. Silicone has been reported to trigger a variety of inflammatory and immunological (both humoral and cellular) responses in humans [39-42] and experimental animals [43]. Experimentally, plasma cell tumors (peritoneal plasmacytomas) could be induced in up to 80% of genetically susceptible mice with intraperitoneal injection of silicone gels and oils [9,44]. It is unlikely that our inability to detect significantly increased numbers of paraproteinemia cases in conjunction with prior silicone implants in women with CTD was related to inadequate exposure to silicone. The 15.4-year mean duration of silicone implant exposure (median 15.8 years, range 0.9–31.3 years) in our case–control study approached the exposure time of women with previously diagnosed CTD and MGUS/MM (mean 17.4 years, median 16.8 years, range 10.2–29.0 years), and both groups had a high rate of implant rupture or leak (≥52% and ≥71%, respectively).

Unexpected findings were the significantly lower serum total protein and α_1_-globulin, α_2_-globulin, β-globulin, γ-globulin, and IgG levels in those with silicone implants compared with those without silicone implants, in both the presence and absence of CTD. We have found no comprehensive study of the serum protein profile in silicone implants subjects in the literature, and the results reported for selected serum proteins and/or protein fractions are conflicting. Hypergammaglobulinemia has been reported in both women with silicone implants [11,45] and in mouse models of silicone exposure [9,46]. In contrast, the total gammaglobulin levels in 2,721 consecutive women with silicone implants [47], and the IgG, IgA and IgM levels in 156 women with silicone implants and rheumatic disease complaints [48], were found to be normal. Furthermore, the proportions of increased or decreased IgG, IgA, and IgM levels between well-matched groups of 298 'healthy' women with and without breast implants were similar in the Women's Health Study [14]. Our findings of lower γ-globulin and immunoglobulin levels in women with silicone implants compared with those in women without implants are thus at variance with these earlier observations. Since all serum globulin fractions tended to be lower with silicone implant exposure in both subjects with various CTD and in healthy subjects, CTD is an unlikely contributor. The etiology of this possible effect of silicone implants remains unclear and requires further study.

Limitations of our study include comparatively small sample sizes, heterogeneity of women regarding the type and length of their silicone implant exposure and CTD diagnoses, lack of quantitative information regarding markers of autoimmune diseases, incomplete information regarding possible treatment effects (type and dose of medications), lack of data for the possible presence of abnormal urinary free light chains, unavailability of bone marrow studies, and lack of extended follow-up regarding the possible development of additional paraproteins and/or possible conversion into MM.

## Conclusion

We found unexpected significant differences in the serum protein profiles of women with silicone implants compared with those without silicone implants, but no evidence for a substantially increased risk of paraproteinemia. From a public health point of view, silicone implants appear to have a minimal, if any, effect on the number of women in whom paraproteins may occur, even in the context of coexisting connective tissue disease.

## Abbreviations

CTD = connective tissue disease; FDA = US Food and Drug Administration; FMS = fibromyalgia syndrome; MGUS = monoclonal gammopathy of undetermined significance; MM = multiple myeloma; NIH = National Institutes of Health; UCTD = undifferentiated connective tissue disease.

## Competing interests

The authors declare that they have no competing interests.

## Authors' contributions

FWM, GC, DJC, HJW, TPO, and EAS designed the study. GC, FWM, RC, EAS, AT, TPO, DJC, and HJW acquired the data. GC, FWM, RC, and AT analyzed and interpreted the data. GC and FWM prepared the manuscript. GC, FWM, and RC performed the statistical analysis.
